# Supplementary material for: Analysis of Control Characteristics between Dominant and Non-Dominant Hands by Transient Responses of Circular Tracking Movements in 3D Virtual Reality Space
Source: Sensors (Basel). 2020 Jun 19;20(12):3477. doi: 10.3390/s20123477 (PMC7348742; doi:10.3390/s20123477)
Supplement: Supplementary file 1 [file sensors-20-03477-s001.pdf]

Supplementary

Table 1. A Summary of Statistical Analysis of *Ipv2* For the Circular Tracking Movement.

Item	Variable	Test	Statistic	Confidence
A	*IPV2* between the dominant and non-dominant hands at each target speed	Two-way repeated measures ANOVA	hand: Mauchly's Test $\chi^2(0) = 0$, p = Nothing, $\varepsilon = 1$; $F\ (1,28) = 0.386$; speed: Mauchly's Test $\chi^2(5) = 10.496$, $p = 0.063$, $\varepsilon = 0.808$; $F\ (3,84) = 32.983$; interaction: Mauchly's Test $\chi^2(5) = 21.667$, $p = 0.001$, $\varepsilon = 0.715$; $F\ (2.146,60.084) = 2.933$	hand: $p = 0.540$, *partial* $\eta^2 = 0.014$, power = 0.092, corrected by Huynh-Feldt; speed: $p = 0$, *partial* $\eta^2 = 0.541$, power = 1.0, corrected by Sphericity Assumed; interaction: $p = 0.057$, *partial* $\eta^2 = 0.095$, power = 0.571, corrected by Greenhouse-Geisser
B	*IPV2* under the conditions of *S1*, *S2*, *S3*, *S4* between *DH* and *NDH*	Bonferroni-corrected pairwise comparisons	*S1* between *DH* and *NDH*: $t\ (28) = 1.704$; *S2* between *DH* and *NDH*: $t\ (28) = 1.037$; *S3* between *DH* and *NDH*: $t\ (28) = 1.433$; *S4* between *DH* and *NDH*: $t\ (28) = 1.369$	*S1* between *DH* and *NDH*: $p = 0.099$, $CI = -43.241 \sim 3.964$, $r = 0.307$; *S2* between *DH* and *NDH*: $p = 0.308$, $CI = -33.826 \sim 11.085$, $r = 0.192$; *S3* between *DH* and *NDH*: $p = 0.163$, $CI = -5.144 \sim 29.075$, $r = 0.261$; *S4* between *DH* and *NDH*: $p = 0.182$,

				CI = -2.707 ~ 13.610, *r* = 0.250
C	*IPV2* of target speeds under the conditions of *S1* : *S2*, *S1* : *S3*, *S1* : *S4*, *S2* : *S3*, *S2* : *S4*, *S3* : *S4* on the *DH* phase	Bonferroni-corrected pairwise comparisons	*S1*: *S2*: *t* (28) = 1.814; *S1*: *S3*: *t* (28) = 2.233; *S1*: *S4*: *t* (28) = 4.661; *S2*: *S3*: *t* (28) = 1.429; *S2: S4*: *t* (28) = 4.060; *S3*: *S4*: *t* (28) = 2.225	*S1*: *S2*; *p* = 0.482, *CI* = -6.482 ~ 29.444, *r* = 0.324; *S1*: *S3*: *p* = 0.202, *CI* = -6.360 ~ 53.258, *r* = 0.389; *S1*: *S4*: *p* = 0, *CI* = 14.740 ~ 60.663, *r* = 0.661; *S2*: *S3*: *p* = 0.985, *CI* = -11.811 ~ 35.748, *r* = 0.261; *S2: S4*: *p* = 0.002, *CI* = 7.887 ~ 44.555, *r* = 0.609; *S3*: *S4*: *p* = 206, *CI* = -3.934 ~ 32.439, *r* = 0.388
D	*IPV2* of target speeds under the conditions of *S1* : *S2*, *S1* : *S3*, *S1* : *S4*, *S2* : *S3*, *S2* : *S4*, *S3* : *S4* on the *NDH* phase	Bonferroni-corrected pairwise comparisons	*S1*: *S2*: *t* (28) = 1.719; *S1*: *S3*: *t* (28) = 5.452; *S1*: *S4*: *t* (28) = 7.213; *S2*: *S3*: *t* (28) = 5.021; *S2*: *S4*: *t* (28) = 5.561; *S3*: *S4*: *t* (28) = 1.548	*S1*: *S2*: *p* = 0.580, *CI* = -12.873 ~ 52.370, *r* = 0.309; *S1*: *S3*: *p* = 0, *CI* = 26.386 ~ 83.721, *r* = 0.718; *S1*: *S4*: *p* = 0, *CI* = 38.077 ~ 87.507, *r* = 0.806; *S2*: *S3*: *p* = 0, *CI* = 15.343 ~ 55.267, *r* = 0.688; *S2*: *S4*: *p* = 0, *CI* = 21.070 ~ 65.016, *r* = 0.724; *S3*: *S4*: *p* = 0.798, *CI* = -6.456 ~ 21.933, *r* = 0.281
E	*IPV2* of target speeds under the conditions of *S1* : *S2*, *S1* : *S3*, *S1* : *S4*, *S2* : *S3*, *S2* : *S4*, *S3* : *S4* on both the *DH* and *NDH* phases	Bonferroni-corrected pairwise comparisons	*S1*: *S2*: *t* (28) = 2.396; *S1*: *S3*: *t* (28) = 6.074; *S1*: *S4*: *t* (28) = 7.995;	*S1*: *S2*: *p* = 0.141, *CI* = -2.886 ~ 34.115, *r* = 0.412; *S1*: *S3*: *p* = 0, *CI* = 20.907 ~ 57.596, *r* = 0.754;

			S2: *S3*: *t* (28) = 4.524; *S2*: *S4*: *t* (28) = 7.455; *S3*: *S4*: *t* (28) = 2.837	*S1*: *S4*: *p* = 0, *CI* = 32.405 ~ 68.089, *r* = 0.834; *S2*: *S3*: *p* = 0.001, *CI* = 8.806 ~ 38.468, *r* = 0.650; *S2*: *S4*: *p* = 0, *CI* = 21.445 ~ 47.820, *r* = 0.815; *S3*: *S4*: *p* = 0.050, *CI* = -0.008 ~ 21.999, *r* = 0.472

Supplementary

Table 2. A Summary of Statistical Analysis of *IPT2* for the Circular Tracking Movement.

Item	Variable	Test	Statistic	Confidence
A	*IPT2* between the dominant and non-dominant hands at each target speed	Two-way repeated measures ANOVA	hand: Mauchly's Test $\chi^2(0) = 0$, p = Nothing, $\varepsilon = 1$; $F(1,28) = 6.276$; speed: Mauchly's Test $\chi^2(5) = 7.874$, $p = 0.164$, $\varepsilon = 0.830$; $F(3,84) = 1.098$; interaction: Mauchly's Test $\chi^2(5) = 9.539$, $p = 0.090$, $\varepsilon = 0.820$; $F(3,84) = 1.123$	hand: $p = 0.018$, *partial* $\eta^2 = 0.183$, power = 0.677, corrected by Huynh-Feldt; speed: $p = 0.354$, *partial* $\eta^2 = 0.038$, power = 0.287, corrected by Sphericity Assumed; interaction: $p = 0.345$, *partial* $\eta^2 = 0.039$, power = 0.293, corrected by Sphericity Assumed

B	*IPT2* under the conditions of *S1*, *S2*, *S3*, *S4* between *DH* and *NDH*	Bonferroni-corrected pairwise comparisons	*S1* between *DH* and *NDH*: t (28) = 1.947;<br><br>*S2* between *DH* and *NDH*: t (28) = 0.677;<br><br>*S3* between *DH* and *NDH*: t (28) = 0.514;<br><br>*S4* between *DH* and *NDH*: t (28) = 2.019	*S1* between *DH* and *NDH*: p = 0.062,<br>*CI* = -0.137 ~ 0.003, r = 0.345;<br><br>*S2* between *DH* and *NDH*: p = 0.504,<br>*CI* = -0.071 ~ 0.036, r = 0.127;<br><br>*S3* between *DH* and *NDH*: p = 0.611,<br>*CI* = -0.065 ~ 0.039, r = 0.097;<br><br>*S4* between *DH* and *NDH*: p = 0.053,<br>*CI* = -0.145 ~ 0.001, r = 0.356
C	*IPT2* of target speeds under the conditions of *S1* : *S2*, *S1* : *S3*, *S1* : *S4*, *S2* : *S3*, *S2* : *S4*, *S3* : *S4* on the *DH* phase	Bonferroni-corrected pairwise comparisons	*S1*: *S2*: t (28) = 1.652;<br>*S1*: *S3*: t (28) = 2.371;<br>*S1*: *S4*: t (28) = 0.777;<br><br>*S2*: *S3*: t (28) = 0.638;<br>*S2: S4*: t (28) = 1.327;<br><br>*S3*: *S4*: t (28) = 1.812	*S1*: *S2*; p = 0.658,<br>*CI* = -0.133 ~ 0.035, r = 0.298;<br>*S1*: *S3*: p = 0.149,<br>*CI* = -0.144 ~ 0.013, r = 0.409;<br>*S1*: *S4*: p = 1, *CI* = -0.112 ~ 0.064, r = 0.145;<br><br>*S2*: *S3*: p = 1, *CI* = -0.090 ~ 0.057, r = 0.120;<br>*S2: S4*: p = 1, *CI* = -0.028 ~ 0.078, r = 0.243;<br><br>*S3*: *S4*: p = 0.485,<br>*CI* = -0.023 ~ 0.106, r = 0.324
D	*IPT2* of target speeds under the conditions of *S1* : *S2*, *S1* : *S3*, *S1* : *S4*, *S2* : *S3*, *S2* : *S4*, *S3* : *S4* on the *NDH* phase	Bonferroni-corrected pairwise comparisons	*S1*: *S2*: t (28) = 0;<br>*S1*: *S3*: t (28) = 0.406;<br>*S1*: *S4*: t (28) = 0.694;	*S1*: *S2*: p = 1, *CI* = -0.090 ~ 0.090, r = 0.000;<br>*S1*: *S3*: p = 1, *CI* = -0.095 ~ 0.071, r = 0.077;

			S2: *S3*: *t* (28) = 0.629;<br>*S2*: *S4*: *t* (28) = 0.755;<br>*S3*: *S4*: *t* (28) = 0.485	*S1*: *S4*: *p* = 1, *CI* = -0.150 ~ 0.091, *r* = 0.130;<br>*S2*: *S3*: *p* = 1, *CI* = -0.065 ~ 0.042, *r* = 0.118;<br>*S2*: *S4*: *p* = 1, *CI* = -0.140 ~ 0.081, *r* = 0.141;<br>*S3*: *S4*: *p* = 1, *CI* = -0.121 ~ 0.086, *r* = 0.091
E	*IPT2* of target speeds under the conditions of *S1* : *S2*, *S1* : *S3*, *S1* : *S4*, *S2* : *S3*, *S2* : *S4*, *S3* : *S4* on both the *DH* and *NDH* phases	Bonferroni-corrected pairwise comparisons	*S1*: *S2*: *t* (28) = 1.060;<br>*S1*: *S3*: *t* (28) = 1.683;<br>*S1*: *S4*: *t* (28) = 0.976;<br>*S2*: *S3*: *t* (28) = 0.857;<br>*S2*: *S4*: *t* (28) = 0.112;<br>*S3*: *S4*: *t* (28) = 0.604	*S1*: *S2*: *p* = 1, *CI* = -0.090 ~ 0.041, *r* = 0.196;<br>*S1*: *S3*: *p* = 0.620, *CI* = -0.104 ~ 0.027, *r* = 0.303;<br>*S1*: *S4*: *p* = 1, *CI* = -0.105 ~ 0.051, *r* = 0.181;<br>*S2*: *S3*: *p* = 1, *CI* = -0.061 ~ 0.033, *r* = 0.160;<br>*S2*: *S4*: *p* = 1, *CI* = -0.059 ~ 0.055, *r* = 0.022;<br>*S3*: *S4*: *p* = 1, *CI* = -0.044 ~ 0.068, *r* = 0.113

Supplementary

Table 3. A Summary of Statistical Analysis of *TD2* for the Circular Tracking Movement.

Item	Variable	Test	Statistic	Confidence
A	*TD2* between the dominant and non-dominant hands at each target speed	Two-way repeated measures ANOVA	hand: Mauchly's Test $\chi^2(0) = 0$, p = Nothing, $\varepsilon = 1$; F (1,28) = 20.310; speed: Mauchly's Test $\chi^2(5) = 5.081$, $p = 0.406$, $\varepsilon = 0.888$; F (3,84) = 15.392; interaction: Mauchly's Test $\chi^2(5) = 7.612$, $p = 0.179$, $\varepsilon = 0.857$; F (3,84) = 3.203	hand: $p = 0$, *partial* $\eta^2 = 0.420$, power = 0.992, corrected by Huynh-Feldt; speed: $p = 0$, *partial* $\eta^2 = 0.355$, power = 1.0, corrected by Sphericity Assumed; interaction: $p = 0.027$, *partial* $\eta^2 = 0.103$, power = 0.720, corrected by Sphericity Assumed
B	*TD2* under the conditions of *S1*, *S2*, *S3*, *S4* between *DH* and *NDH*	Bonferroni-corrected pairwise comparisons	*S1* between *DH* and *NDH*: t (28) = 3.964; *S2* between *DH* and *NDH*: t (28) = 4.824; *S3* between *DH* and *NDH*: t (28) = 1.091; *S4* between *DH* and *NDH*: t (28) = 1.764	*S1* between *DH* and *NDH*: $p = 0$, CI = -0.075 ~ -0.024, $r = 0.600$; *S2* between *DH* and *NDH*: $p = 0$, CI = -0.101 ~ -0.041, $r = 0.674$; *S3* between *DH* and *NDH*: $p = 0.285$, CI = -0.043 ~ -0.013, $r = 0.202$;

				S4 between *DH* and *NDH*: *p* = 0.089, *CI* = -0.069 ~ -0.005, *r* = 0.316
C	*TD2* of target speeds under the conditions of *S1* : *S2*, *S1* : *S3*, *S1* : *S4*, *S2* : *S3*, *S2* : *S4*, *S3* : *S4* on the *DH* phase	Bonferroni-corrected pairwise comparisons	*S1*: *S2*: *t* (28) = 3.393; *S1*: *S3*: *t* (28) = 2.615; *S1*: *S4*: *t* (28) = 3.371; *S2*: *S3*: *t* (28) = 0.205; *S2: S4*: *t* (28) = 0.916; *S3*: *S4*: *t* (28) = 1.173	*S1*: *S2*; *p* = 0.014, *CI* = 0.006 ~ 0.075, *r* = 0.540; *S1*: *S3*: *p* = 0.085, *CI* = -0.003 ~ 0.078, *r* = 0.443; *S1*: *S4*: *p* = 0.014, *CI* = 0.009 ~ 0.105, *r* = 0.537; *S2*: *S3*: *p* = 1, *CI* = -0.051 ~ 0.044, *r* = 0.039; *S2: S4*: *p* = 1, *CI* = -0.034 ~ 0.066, *r* = 0.171; *S3*: *S4*: *p* = 1, *CI* = -0.028 ~ 0.067, *r* = 0.216
D	*TD2* of target speeds under the conditions of *S1* : *S2*, *S1* : *S3*, *S1* : *S4*, *S2* : *S3*, *S2* : *S4*, *S3* : *S4* on the *NDH* phase	Bonferroni-corrected pairwise comparisons	*S1*: *S2*: *t* (28) = 1.704; *S1*: *S3*: *t* (28) = 5.750; *S1*: *S4*: *t* (28) = 5.255; *S2*: *S3*: *t* (28) = 5.070; *S2*: *S4*: *t* (28) = 4.108; *S3*: *S4*: *t* (28) = 0.239	*S1*: *S2*: *p* = 0.597, *CI* = -0.013 ~ 0.051, *r* = 0.306; *S1*: *S3*: *p* = 0, *CI* = 0.036 ~ 0.107, *r* = 0.736; *S1*: *S4*: *p* = 0, *CI* = 0.034 ~ 0.114, *r* = 0.705; *S2*: *S3*: *p* = 0, *CI* = 0.023 ~ 0.082, *r* = 0.692; *S2*: *S4*: *p* = 0.002, *CI* = 0.017 ~ 0.093, *r* = 0.613; *S3*: *S4*: *p* = 1, *CI* = -0.029 ~ 0.034, *r* = 0.045
E	*TD2* of target speeds under the conditions of *S1* : *S2*, *S1* : *S3*, *S1* :	Bonferroni-corrected pairwise comparisons	*S1*: *S2*: *t* (28) = 3.487;	*S1*: *S2*: *p* = 0.010, *CI* = 0.005 ~ 0.051, *r* = 0.550;

S4, *S2* : *S3*, *S2* : *S4*, *S3* : *S4* on both the *DH* and *NDH* phases	*S1*: *S3*: t (28) = 4.861;<br>*S1*: *S4*: t (28) = 5.406;<br>*S2*: *S3*: t (28) = 2.515;<br>*S2*: *S4*: t (28) = 3.382;<br>*S3*: *S4*: t (28) = 1.090	*S1*: *S3*: p = 0, *CI* = 0.023 ~ 0.086, r = 0.677;<br>*S1*: *S4*: p = 0, *CI* = 0.031 ~ 0.100, r = 0.715;<br>*S2*: *S3*: p = 0.108, *CI* = -0.003 ~ 0.052, r = 0.429;<br>*S2*: *S4*: p = 0.014, *CI* = 0.005 ~ 0.066, r = 0.539;<br>*S3*: *S4*: p = 1, *CI* = -0.018 ~ 0.040, r = 0.202
